# When Should I Be Associating a Lateral Extra‐Articular Procedure to My Anterior Cruciate Ligament Reconstruction? AI vs. Surgeon Decision‐Making

**DOI:** 10.1155/aort/8238794

**Published:** 2025-12-14

**Authors:** Simone Giusti, Marco Susca, Andreas Luchetti, Salvatore Congiusta, Ezio Adriani

**Affiliations:** ^1^ Complex Operational Unit of Sports Medicine and Joint Reconstruction, Fondazione Policlinico Universitario Agostino Gemelli IRCCS, Rome, Italy; ^2^ Department of Geriatrics, Orthopaedics, and Rheumatology, Università Cattolica del Sacro Cuore, Milano, Italy, unicatt.it; ^3^ Complex Operational Unit of Trauma and Orthopaedics, Fondazione Policlinico Universitario Campus Bio-Medico, Rome, Italy

## Abstract

**Purpose:**

Residual rotational instability following anterior cruciate ligament reconstruction (ACLR) remains a clinical challenge, leading to renewed interest in adjunctive lateral extra‐articular procedures (LEAP). This study aimed to compare clinical decision‐making between experienced orthopaedic surgeons and an artificial intelligence (AI) model regarding indications for LEAP in ACLR, to assess concordance, and to explore the potential role of AI in surgical planning.

**Methods:**

A cross‐sectional comparative study was conducted using 40 hypothetical ACLR case profiles, reflecting a range of patient demographics, injury characteristics, and activity levels. An AI model trained on literature‐based criteria and expert input generated binary recommendations (“perform LEAP” or “do not perform LEAP”) for each case. Twenty‐two high‐volume knee surgeons independently reviewed all cases, blinded to AI recommendations, and indicated whether they would recommend a LEAP. Agreement between surgeon decisions and AI recommendations was calculated, and factors influencing concordance were analysed using chi‐square tests, *t*‐tests, and Pearson correlations (*p* < 0.05).

**Results:**

Overall, surgeon agreement with AI recommendations was high but varied by clinical factors. A positive pivot shift test was the strongest predictor of concordance (93.9% ± 4.8 vs. 71.8% ± 25.7; *p* = 0.0004, Cohen’s *d* = 1.23). Surgeons agreed more often when the AI recommended LEAP (92.7% ± 6.5) than when it advised against it (70.9% ± 27.0; *p* = 0.0006). Male patient cases yielded higher agreement (91.1% ± 7.4) compared with female cases (75.7% ± 27.0; *p* = 0.018). Ligamentous laxity (Beighton score) showed a moderate positive correlation with agreement (*r* = 0.39; *p* = 0.013), while age, revision status, associated lesions, and time from injury to surgery were not significant predictors.

**Conclusion:**

Surgeons demonstrated strong alignment with AI recommendations in clear‐cut scenarios, particularly when traditional clinical signs such as a positive pivot shift were present. Discordance emerged in borderline cases, notably when AI recommended against LEAP or in female patients. These findings suggest AI could support orthopaedic decision‐making by standardising criteria for LEAP, enhancing consistency in ambiguous cases, and prompting the development of evidence‐based scoring systems to refine indications.

**Trial Registration:**

IRB cleared, no need for trial registration as all cases hypothetical and no patient data included in the study.

## 1. Introduction

Anterior cruciate ligament (ACL) tears are among the most common and functionally debilitating injuries in active populations, particularly athletes participating in pivoting sports. ACL reconstruction (ACLR) remains the gold standard surgical intervention for restoring anterior knee stability and enabling return to high‐level physical activity [1]. However, despite advances in surgical techniques, a subset of patients continue to experience residual rotational instability following isolated ACLR—particularly those with high‐grade pivot shifts, generalized ligamentous laxity, or involvement in high‐demand sports [2].

To address this, there has been renewed growing interest in lateral extra‐articular articular procedures (LEAP), as adjuncts to intra‐articular ACLR, as demonstrated by the recent consensus meeting. Originally developed prior to the modern era of arthroscopy, LEAP involves reinforcing the anterolateral aspect of the knee—originally using a strip of the iliotibial band—to provide an external mechanical restraint against internal tibial rotation [3]. Recent studies suggest that LEAP, when selectively indicated, may improve rotational stability, reduce graft failure rates, and enhance return‐to‐sport outcomes [4, 5] without significantly compromising knee kinematics or increasing complication rates [6–8].

Despite growing evidence supporting LEAP in high‐risk cohorts, the clinical decision‐making process for selecting appropriate candidates remains variable, relying heavily on surgeon experience, intraoperative findings, and subjective clinical assessment [9]. In this context, artificial intelligence (AI) has emerged as a potential tool to standardize and support clinical decision‐making. With increasing integration of AI algorithms in orthopaedics, the possibility of using AI to assist in surgical indication and planning warrants rigorous evaluation.

The purpose of this study is to compare the clinical indications for ACLR with concomitant LEAP as determined by experienced orthopaedic surgeons versus an AI‐based decision‐making protocol. By contrasting human expert judgement with algorithmic recommendations, this investigation aims to explore the potential role of AI in facilitating complex orthopaedic decision‐making processes in what still is an extremely debated topic amongst experienced surgeons.

This study is important because it addresses variability in surgical indications for LEAP in ACL reconstruction. By incorporating AI, our work proposes a structured decision‐support tool that can be integrated into the current decision‐making scenario, where clinical equipoise persists. We hypothesized that AI‐based support could reduce intersurgeon variability in LEAP indications and provide a reproducible framework for clinical decision‐making.

## 2. Methods

### 2.1. Study Design

We carried out a cross‐sectional, comparative analysis designed to evaluate agreement between AI recommendations and senior orthopaedic surgeon (fellowship‐trained in sports medicine) decision‐making regarding the use of LEAP in association with ACLR. The primary aim was to identify factors influencing surgeon agreement with AI‐based recommendations for LEAP.

### 2.2. Case Dataset

A total of 40 unique, hypothetical clinical ACLR case profiles were selected for inclusion. Each case was derived from anonymized patient data representing a wide spectrum of clinical scenarios relevant to ACL reconstruction, including variations in patient age, sex, activity level, sport participation, generalized ligamentous laxity, time from injury, presence of high‐grade pivot shift and associated lesions (meniscal and cartilage). No imaging parameters were employed for case creation, as the focus was on clinical decision‐making. All of the cases were hypothetical and not related to any real‐life patient scenarios; therefore, institutional review board (IRB) exemption was obtained due to the lack of patient‐specific data.

### 2.3. AI Recommendation Model

An AI model trained on a curated dataset of expert surgeon decisions and relevant literature‐derived criteria was used to generate binary recommendations (“Perform LEAP” or “Do Not Perform LEAP”) for each of the 40 cases. The model was developed using supervised learning with prior validation on a hold‐out set to ensure clinical logic consistency. To prevent overfitting, early stopping and regularization were applied. Future iterations will include larger datasets and external validation. Model variables included demographics, biomechanical risk factors, and imaging‐based parameters. The AI system served solely as a recommendation tool and did not adjust based on surgeon input.

### 2.4. Surgeon Panel

Twenty‐two senior, high‐volume (> 200 ACL reconstructions per year with over 15 years of clinical practice) orthopaedic surgeons specializing in sports medicine and knee ligamentous surgery participated in the study. All participants were blinded to AI recommendations at the time of their assessment. Each surgeon independently reviewed all 40 clinical cases and was asked to decide whether they would recommend a LEAP procedure in conjunction with ACLR. No time limits were imposed, and responses were collected using a secure, web‐based survey platform.

### 2.5. Outcome Measures

The primary outcome was the surgeon agreement rate with the AI recommendation for each case, calculated as the percentage of surgeons whose decision matched the AI output. A case was considered “AI‐concordant” if at least 50% of the surgeons agreed with the AI recommendation. Secondary outcomes included analysis of factors associated with surgeon‐AI agreement at both the case and surgeon levels.

### 2.6. Statistical Analysis

All statistical analyses were conducted using SPSS (Version XX, IBM Corp, Armonk, NY). Categorical variables were compared using chi‐square (*χ* ^2^) tests. Continuous variables were compared using independent *t*‐tests. Correlations between surgeon agreement rates and continuous case features were assessed using Pearson correlation coefficients. Effect sizes were calculated using Cohen’s *d* for mean differences and *r* for correlation strengths. A significance threshold of *p* < 0.05 was used for all comparisons.

## 3. Results

The analysis revealed a statistically significant association between patient sex and surgeon agreement with AI recommendations (*p* = 0.018). Male patients demonstrated consistently higher agreement rates compared to female patients, with males achieving a mean agreement rate of 91.1% (SD = 7.4%, *n* = 25) versus females at 75.7% (SD = 27.0%, *n* = 15). The chi‐square test yielded a test statistic of *χ* ^2^ = 5.56, indicating a significant association. The effect size, measured by Cohen’s *d*, was 0.78, representing a large effect according to conventional interpretations (Cohen’s *d* ≥ 0.8 indicates a large effect). The pivot shift test emerged as the strongest predictor of surgeon agreement, showing highly significant results (*p* = 0.0004). Cases with positive pivot shift tests achieved remarkably high agreement rates of 93.9% (SD = 4.8%, *n* = 18), while cases with negative pivot shift tests showed considerably lower agreement at 71.8% (SD = 25.7%, *n* = 22). The chi‐square statistic was *χ* ^2^ = 12.67, and the effect size of Cohen’s *d* = 1.23 represents a very large effect, exceeding the threshold for large effects. The standard deviation difference is particularly noteworthy: positive cases showed minimal variability (SD = 4.8%), indicating near‐universal agreement among surgeons, while negative cases demonstrated substantial variability (SD = 25.7%), suggesting considerable disagreement in these scenarios.

The distinction between primary and revision ACL reconstruction did not significantly influence surgeon agreement (*p* = 0.275). Primary cases showed agreement rates of 86.4% (SD = 16.8%, *n* = 25), while revision cases achieved 78.7% (SD = 24.4%, *n* = 15). The chi‐square statistic was *χ* ^2^ = 1.19, with a small‐to‐medium effect size of Cohen’s *d* = 0.38. The lack of statistical significance suggests that revision status does not substantially impact surgeon consensus regarding LEAP recommendations.

The presence and type of associated lesions (medial meniscus, lateral meniscus, chondral lesions, or none) did not significantly affect surgeon agreement (*p* = 0.270). One‐way ANOVA yielded *F* = 1.36, indicating no significant differences among groups. Similarly, patient age showed no significant correlation with surgeon agreement (*r* = −0.14, *p* = 0.381). The mean age was 26.4 years (SD = 8.2 years, range 14–41 years). Interestingly, patients in cases where AI recommended LEAP were significantly younger (mean = 21.8 years, SD = 6.4) compared to those where AI recommended against LEAP (mean = 32.7 years, SD = 5.9), with a *t*‐test yielding *t* = −5.45, *p* < 0.001, and a very large effect size of Cohen’s *d* = −1.74. This age difference reflects the AI’s consideration of activity level and long‐term outcomes in younger patients, but age itself does not significantly influence surgeon agreement with these recommendations.

Furthermore, the time interval from injury to surgery showed no correlation with surgeon agreement (*r* = −0.006, *p* = 0.971). The mean interval was 109.4 days (SD = 122.6 days, range 5–450 days). Similar to age, there was a significant difference between AI recommendation groups: cases where AI recommended LEAP had shorter intervals (mean = 47.6 days, SD = 68.9) compared to cases where AI recommended against LEAP (mean = 192.9 days, SD = 131.3), with *t* = −4.54, *p* = 0.0001, and Cohen’s *d* = −1.45. This suggests the AI considers timing in its recommendations, but surgeons’ agreement is not influenced by this temporal factor.

The Tegner Activity Scale, measuring preinjury activity level, showed no significant correlation with surgeon agreement (*r* = 0.12, *p* = 0.445). The mean Tegner score was 5.8 (SD = 1.9, range 2–9). Cases where AI recommended LEAP had significantly higher activity levels (mean = 6.9, SD = 1.4) compared to those where AI recommended against LEAP (mean = 4.4, SD = 1.4), with *t* = 5.67, *p* < 0.001, and Cohen’s *d* = 1.81. This very large effect size indicates that activity level strongly influences AI recommendations, but surgeon agreement remains independent of this factor.

Ligament laxity, measured by the Beighton hypermobility score, demonstrated a significant moderate positive correlation with surgeon agreement (*r* = 0.39, *p* = 0.013). The mean Beighton score was 2.6 (SD = 1.8, range 0–6). This represents the only continuous variable significantly associated with surgeon agreement. Higher Beighton scores, indicating greater joint hypermobility, were associated with higher surgeon agreement rates. Cases where AI recommended LEAP had higher Beighton scores (mean = 3.0, SD = 1.7) compared to those where AI recommended against LET (mean = 2.0, SD = 1.9), though this difference was marginally significant (*t* = 1.79, *p* = 0.082, Cohen’s *d* = 0.57).

Lastly, the direction of the AI recommendation itself significantly influenced surgeon agreement (*p* = 0.0006). Cases where the AI recommended LEAP (Yes) achieved high agreement rates of 92.7% (SD = 6.5%, *n* = 23), while cases where the AI recommended against LET (No) showed lower agreement at 70.9% (SD = 27.0%, *n* = 17). The chi‐square test statistic was *χ* ^2^ = 11.88, with a very large effect size of Cohen’s *d* = 1.20 for LEAP. These results are summarised in Table 1.

**Table 1:** Summary of results.

Variable	Groups	Mean agreement (%) ± SD	*p* value
Patient sex	Male (*n* = 25)	91.1 ± 7.4	0.018
	Female (*n* = 15)	75.7 ± 27.0	

Pivot shift test	Positive (*n* = 18)	93.9 ± 4.8	0.0004
	Negative (*n* = 22)	71.8 ± 25.7	

AI recommendation	LET recommended (*n* = 23)	92.7 ± 6.5	0.0006
	LET not recommended (*n* = 17)	70.9 ± 27.0	

Surgery type	Primary (*n* = 25)	86.4 ± 16.8	0.275
	Revision (*n* = 15)	78.7 ± 24.4	

Associated lesions	Medial meniscus (*n* = 7)	94.2 ± 4.3	0.270
	Lateral meniscus (*n* = 3)	89.4 ± 14.6	
	Chondral lesion (*n* = 9)	86.9 ± 5.8	
	None (*n* = 21)	77.5 ± 27.1	

Patient age	Continuous	*r* = −0.14	0.381

Days injury to surgery	Continuous	*r* = −0.006	0.971

Tegner activity scale	Continuous	*r* = 0.12	0.445

Beighton score	Continuous	*r* = 0.39	0.013

The higher agreement with AI recommendations to perform LEAP may reflect a cognitive bias toward intervention in high‐risk patients. This aligns with known decision‐making biases in medicine, where action is often favored over omission in the face of potential risk.

## 4. Discussion

This study presents a novel comparison between AI‐driven recommendations and orthopedic surgeons’ consensus regarding the indication for LEAP in ACL reconstruction. Our findings demonstrate that while overall agreement with AI is high, significant variability exists depending on clinical variables, particularly sex, pivot shift results, and the AI recommendation direction itself.

It was found that male patients had a significantly higher agreement rate (91.1%) among surgeons compared to female patients (75.7%). Although sex‐based differences in ACL injury biomechanics and outcomes have been widely documented, few studies have addressed whether sex influences decision‐making consistency for surgical augmentation procedures [10]. This discrepancy may reflect surgeon biases or uncertainty in interpreting injury risk profiles and laxity patterns in female patients. The larger utility of associating LEAP in the male population has also been discussed by several studies who consider males to be at greater risk of residual instability compared to females [11].

The pivot shift test was the strongest single predictor of agreement, with 93.9% agreement for positive tests compared to 71.8% for negative tests. This aligns with previous literature emphasizing the role of pivot shift as a clinical marker of rotational instability, which is a key indication for LEAP. For instance, Sonnery‐Cottet et al. [12] emphasized the utility of the pivot shift in predicting failure risk in high‐demand patients’ post‐ACL reconstruction. The low variability in surgeon agreement in the presence of a positive pivot shift underscores its clinical reliability.

Interestingly, surgeon agreement was substantially higher when AI recommended in favor of LEAP (92.7%) versus against it (70.9%). This discrepancy might reflect a cognitive bias where clinicians are more inclined to agree with active interventions, especially in younger, athletic populations, where under‐treatment poses greater long‐term risks. It also suggests that while AI can guide clinical practice, surgeon confidence may depend heavily on alignment with interventional strategies. Although not universally recommended, most modern studies indicate that adding a LEAP improves results and reduces the rate of failure​ [13]. However, not all studies advocate for routine LEAP use. A randomized controlled trial found no significant difference in outcomes at 2 years between isolated ACL revision and combined ACL + ALL reconstruction [14]. In addition, concerns remain regarding overconstraint and potential impacts on lateral compartment cartilage, although modern techniques such as the modified Lemaire (a lateral extra‐articular tenodesis involving iliotibial band transfer fixed distal to the lateral femoral epicondyle) procedure aim to minimise these risks [15, 16]. Regarding degenerative changes, recent prospective studies and systematic reviews confirm that combined ACL + LEAP reconstructions do not increase the incidence of lateral compartment osteoarthritis [17].

These aspects were explored in depth in the Stability Study of Getgood et al. [18], a multicenter randomized clinical trial involving over 600 high‐demand athletes, comparing isolated ACLR with ACLR combined with LEAP. At 2 year follow‐up, the addition of LEAP significantly reduced the incidence of clinical graft failure and revision surgery (from 3.5% to 0.7%). However, no significant differences were observed in patient‐reported outcome measures (PROMs) between the two groups, including KOOS, subjective IKDC, and ACL‐RSI scores, suggesting that LEAP does not provide additional benefit in terms of perceived functional outcomes in the short to mid‐term, but enhances rotational stability and reduces the risk of graft failure. This evidence may support the selective use of LEAP in high‐risk patients.

Furthermore, while age, preinjury Tegner score, and time from injury to surgery did not correlate with surgeon agreement, these were significantly different between AI groups. AI tended to recommend LEAP in younger patients with higher Tegner scores and shorter injury‐to‐surgery intervals—consistent with known risk profiles for ACL graft failure [19, 20]. This reflects AI’s sensitivity to high‐risk features, mirroring algorithm‐based predictive models like those by Magnussen et al. [21, 22]. In the recent consensus meeting (2025), in chronic ACL injuries, the addition of a LEAP procedure seemed to provide clinical benefits, recognizing chronicity as a potential indication [23]. However, the lack of surgeon agreement on these parameters suggests that clinical judgement may not fully integrate these quantitative risk indicators.

Ligamentous laxity, as measured by the Beighton score, positively correlated with surgeon agreement (*r* = 0.39, *p* = 0.013) [24]. This moderate correlation indicates that joint hypermobility influences clinician consensus. Hyperlaxity and especially knee hyperextension represent deleterious factors for ACL reconstruction. Patients with > 6.5° hyperextension are at markedly higher risk of graft rupture and worse functional outcomes [25]. Prior studies have linked generalized hyperlaxity with increased ACL failure rates and rotational instability, supporting the use of LEAP in these cases [26–30]. The AI’s marginally significant association with higher Beighton scores aligns with this understanding, reinforcing the predictive validity of AI‐based modelling.

On the contrary, neither revision status nor associated meniscal or chondral lesions significantly influenced surgeon agreement. It is worth noting that some studies suggest that the treatment of meniscal lesions improves pivot shift laxity more than LEAP [31]. Despite previous studies suggesting increased failure risk in revision ACL cases and in those with complex intra‐articular pathology [32], our results indicate that these factors are not consistently weighted in current clinical decision‐making for LEAP.

These findings highlight the potential of AI to standardize decision‐making in ACL surgery by consistently integrating evidence‐based risk factors [33, 34]. Surgeons tend to agree with AI when traditional clinical signs are strong (e.g., positive pivot shift) [35] but diverge in borderline or subjective contexts, particularly when AI recommends against LEAP. This divergence points to a broader need for augmented clinical decision‐support tools that improve surgeon confidence in cases where ambiguity is high.

Importantly, this study suggests that AI may promote more objective surgical planning, especially in young, athletic populations where the risk of failure is high and LEAP is a validated augment. As LEAP continues to gain traction as a prophylactic adjunct in ACL reconstruction—particularly among elite athletes and those with high‐grade pivot shifts—its indication must be precisely defined to avoid overtreatment.

By analysing the consensus between AI and senior surgeons in this study, we therefore suggest a novel weighted points‐based scoring system to aid clinicians in their surgical decision‐making on whether or not to associate an LEAP procedure to an ACL reconstruction. The scoring system is shown in Table 2.

**Table 2:** LET scoring system.

Parameter		Points
Pivot shift test	Positive	10
	Negative	0

Patient sex	Male	10
	Female	0

Age	≤ 25	3
	26–30	1
	> 30	0

Activity level (Tegner scale)	High (> 7)	2
	Moderate (5‐6)	1
	Low (< 5)	0

Time from injury	Acute (≤ 30 days)	2
	Subacute (31–90 days)	1
	Chronic (> 90 days)	0

Surgery type	Primary	1
	Revision	0

Ligament laxity (Beighton score)	High (≥ 4)	1
	Normal (< 4)	0

### 4.1. Score Interpretation

Score ≥ 13: strong recommendation for LEAP.

83% sensitivity, 100% specificity.

Strong surgeon consensus expected.

Score 9–12: Moderate recommendation for LEAP.

Consider patient preferences and surgeon experience.

May benefit from additional evaluation.

Score ≥ 7: Weak recommendation for LEAP.

92% sensitivity, 88% specificity.

Good screening threshold.

Score < 7: LET likely NOT recommended.

Strong consensus against LEAP in most cases.

### 4.2. Limitations

This study has several limitations. First, the sample size of 40 imaginary cases, though reviewed by 22 experienced surgeons, may limit generalizability. Second, while the AI model accounted for multiple clinical features, surgeon decisions likely also reflect unmeasured contextual factors such as institutional protocol, surgeon training, and risk tolerance. Lastly, the AI system’s internal logic was not accessible for transparency, limiting direct comparison. We acknowledge that the current study relies on hypothetical case scenarios. This limits real‐world applicability, and future work should include both prospective and retrospective clinical validation to confirm the reproducibility and external validity of the AI‐assisted decision‐making process.

## 5. Conclusion

AI‐driven recommendations for LEAP in ACL reconstruction align well with surgeon consensus in clearly indicated cases, such as those with positive pivot shifts or joint hyperlaxity. However, discord emerges in ambiguous scenarios, particularly with female patients or when AI recommends against augmentation. These insights support a complementary role for AI in surgical decision‐making, reinforcing objective criteria in cases where surgeon variability remains high.

## Conflicts of Interest

The authors declare no conflicts of interest.

## Funding

No funding was received by any of the authors for the present study.

## Data Availability

Data are available from the corresponding author upon reasonable request.
